# Oligogenic Inheritance Underlying Incomplete Penetrance of *PROKR2* Mutations in Hypogonadotropic Hypogonadism

**DOI:** 10.3389/fgene.2021.665174

**Published:** 2021-09-03

**Authors:** Rahma Mkaouar, Lamia Cherif Ben Abdallah, Chokri Naouali, Saida Lahbib, Zinet Turki, Sahar Elouej, Yosra Bouyacoub, Maali Somai, Kenneth Mcelreavey, Anu Bashamboo, Sonia Abdelhak, Olfa Messaoud

**Affiliations:** ^1^Laboratoire de Génomique Biomédicale et Oncogénétique, Institut Pasteur de Tunis, Tunis, Tunisia; ^2^Faculté des Sciences Mathématiques, Physiques et Naturelles de Tunis, Université de Tunis El Manar, Tunis, Tunisia; ^3^Département d’Endocrinologie et de Technologie Alimentaire, Institut de Nutrition, Tunis, Tunisia; ^4^Génétique du Développement Humain, Institut Pasteur, Paris, France

**Keywords:** hypogonadotropic hypogonadism, Kallmann syndrome, oligogenism, pathogenic combination, digenic score

## Abstract

The role of the prokineticin 2 pathway in human reproduction, olfactory bulb morphogenesis, and gonadotropin-releasing hormone secretion is well established. Recent studies have highlighted the implication of di/oligogenic inheritance in this disorder. In the present study, we aimed to identify the genetic mechanisms that could explain incomplete penetrance in hypogonadotropic hypogonadism (HH). This study involved two unrelated Tunisian patients with HH, which was triggered by identifying a homozygous p.(Pro290Ser) mutation in the *PROKR2* gene in a girl (HH1) with Kallmann syndrome (KS). The functional effect of this variant has previously been well demonstrated. Unexpectedly, her unaffected father (HH1P) and brother (HH1F) also carried this genetic variation at a homozygous state. In the second family, we identified a heterozygous p.(Lys205del) mutation in *PROKR2*, both in a male patient with normosmic idiopathic IHH (HH12) and his asymptomatic mother. Whole-exome sequencing in the three HH1 family members allowed the identification of additional variants in the prioritized genes. We then carried out digenic combination predictions using the oligogenic resource for variant analysis (ORVAL) software. For HH1, we found the highest number of disease-causing variant pairs. Notably, a *CCDC141* variant (c.2803C > T) was involved in 18 pathogenic digenic combinations. The *CCDC141* variant acts in an autosomal recessive inheritance mode, based on the digenic effect prediction data. For the second patient (HH12), prediction by ORVAL allowed the identification of an interesting pathogenic digenic combination between *DUSP6* and *SEMA7A* genes, predicted as “dual molecular diagnosis.” The *SEMA7A* variant p.(Glu436Lys) is novel and predicted as a VUS by Varsome. Sanger validation revealed the absence of this variant in the healthy mother. We hypothesize that disease expression in HH12 could be induced by the digenic transmission of the *SEMA7A* and *DUSP6* variants or a monogenic inheritance involving only the *SEMA7A* VUS if further functional assays allow its reclassification into pathogenic. Our findings confirm that homozygous loss-of-function genetic variations are insufficient to cause KS, and that oligogenism is most likely the main transmission mode involved in Congenital Hypogonadotropic Hypogonadism.

## Introduction

Idiopathic hypogonadotropic hypogonadism (IHH) (MIM ID #146110) is defined as the absence of sex steroid synthesis associated with the lack of appropriate gonadotropin secretion. This leads to a variable degree of impuberism, often diagnosed during childhood or adolescence. When the embryonic migration of gonadotropin-releasing hormone (GnRH) neurons from the nasal placode to their final destination in the hypothalamus is disrupted, the resulting phenotype is Kallmann syndrome (KS), which is defined as the association of HH with hyposmia or anosmia (Sonne and Lopez-Ojeda, 2021). Nineteen genes are known to be involved in KS (*ANOS1*, *FGF8*, *FGFR1*, *FGF17*, *CHD7*, *IL17RD*, *DUSP6*, *SPRY4*, *FLRT3*, *KLB*, *SEMA3A*, *SEMA3E*, *NSMF*, *HS6ST1*, *WDR11*, *SOX10*, *FEZF1*, *IGSF10*, *PROK2*, and *PROKR2*) (Boehm et al., 2015; Stamou and Georgopoulos, 2018). Normosmic idiopathic hypogonadotropic hypogonadism (nIHH), which is not associated with anosmia, and results from the dysfunction of the GnRH neurons that successfully completed their embryonic migration to the hypothalamus. As of today, 46 genes have been associated with IHH (Topaloğlu, 2017; Cangiano et al., 2020). The molecular alterations identified in these genes account for 40% of all IHH cases (Gach et al., 2020), thus suggesting that half of the IHH causal genes remain unknown. Molecular alterations have been identified for several neuropeptides or their corresponding receptors, which are involved in the physiology of the gonadotropic axis: *GNRHR/GNRH1*, *KISS1R/GPR54*, *TAC3/TACR3*, and *PROK2/PROKR2* (Brioude et al., 2010; Topaloglu and Kotan, 2010). In addition to reproductive dysfunction, nIHH/KS patients may also manifest a variety of other non-reproductive disorders, such as midline facial defects, dental agenesis, renal agenesis, hearing loss, or bimanual synkinesis (Young et al., 2019). IHH may be inherited in an X-linked recessive, autosomal dominant, or autosomal recessive modes of inheritance. IHH has been predominantly detected in sporadic cases (Quaynor et al., 2011; Gach et al., 2020).

The *PROK2* gene (NG_008275.1) is located on chromosome 3p21.1 and comprises four exons. The *PROKR2* gene (NG_008132.1) maps to chromosome 20p13 and contains seven transmembrane-domain receptors. Both genes, *PROK2* and *PROKR2*, belong to the family of prokineticins, and a group of multifunctional secreted proteins first identified in 2000 by Li et al. (2001). Their involvement in KS was strongly suggested in 2006 when homozygous *Prokr2* knockout mice were shown to have hypogonadotropic hypogonadism due to hypothalamic GnRH deficiency and agenesis or hypoplasia of the olfactory bulbs (Matsumoto et al., 2006). This was confirmed in the same year by the discovery of genetic variations in patients with KS (Dodé et al., 2006). More than 27 genetic variations in *PROKR2* (Dode and Rondard, 2013) and over 11 genetic variations in *PROK2* have been reported in patients with nIHH or KS (Martin et al., 2011). However, the KS and nIHH genetics are complex and still not well understood (Dode and Hardelin, 2010). In addition, genetic variations in *PROK2* and *PROKR2* genes reported in KS and nIHH patients were found at heterozygous, homozygous, and compound heterozygous states (Dodé et al., 2006; Hardelin and Dode, 2008). Homozygous loss-of-function genetic variations in the *PROK2* gene cause nIHH in mice and humans (Abreu et al., 2008; Schöneberg and Liebscher, 2021). Thus, an autosomal recessive mode of transmission was established (Pitteloud et al., 2007). Later, other studies on different ethnic populations reported a large number of heterozygous genetic variations in *PROK2* and *PROKR2* genes with considerable clinical and molecular heterogeneity among several patients having both KS and nIHH (Cole et al., 2008; Sarfati et al., 2010a). Heterozygous genetic variations inherited from clinically unaffected first-degree relatives in the *PROKR2* gene have been reported in some KS/nIHH patients, which was attributed to digenic/oligogenic transmission rather than a dominant negative effect of monoallelic *PROKR2* genetic variations (Dodé et al., 2006; Abreu et al., 2008; Cole et al., 2008; Monnier et al., 2009; Quaynor et al., 2011; Lewkowitz-Shpuntoff et al., 2012). The hypothesis of oligogenic inheritance postulates that disease expression is induced by more than one mutated IHH gene (Pitteloud et al., 2005; Maione et al., 2018). Indeed the *PROKR2* gene has been involved in several digenic and trigenic associations such as *PROK2/PROKR2*, *FGFR1/PROKR2*, *PROK/GNRHR*, and *PROKR2/CHD7/FEZF1* (Cole et al., 2008; Canto et al., 2009; Sarfati et al., 2010b; Pablo Méndez et al., 2015; Zhang et al., 2020). However, the expression of deleterious alleles is considerably variable if we compare the phenotypes of patients carrying identical variations (Pitteloud et al., 2007; Boehm et al., 2015). Several studies conducted on large cohorts have shown that oligogenic heredity accounts for 2.5–15% of Congenital Hypogonadotropic Hypogonadism (CHH) patients (Cassatella et al., 2018).

Here, we report a clinical and genetic investigation of KS and nIHH in two Tunisian families after excluding the involvement of monogenic inheritance of *PROKR2* gene variants.

## Patients and Methods

### Patients

The current molecular analysis was conducted for two unrelated HH families (HH1—a woman with KS and HH12—a man with nIHH). The index cases were recruited at the Endocrinology Department of the National Institute of Nutrition in Tunis. Both patients belong to simplex families (only one family member is affected, with healthy siblings). For the two patients, GnRH deficiency diagnosis was established based on puberty state (absent or incomplete), hormonal tests (testosterone in HH12 and serum gonadotropins levels), response to GnRH, and anterior pituitary function, which was evaluated by measuring the basal levels of free T4, TSH, and prolactin as well as the cortisol peak levels after ACTH stimulation. Pituitary imaging was performed by magnetic resonance imaging (MRI) to exclude acquired causes of nIHH. Olfactory testing for anosmia or hyposmia was also assessed.

### Molecular Investigation

Genomic DNA was extracted from peripheral blood leucocytes by FlexiGene DNA extraction kit (Qiagen) according to the manufacturer’s instructions. The coding region and intron–exon boundaries of *PROK2* and *PROKR2* genes as well as exon 13 of the *SEMA7A* gene were amplified as previously reported (Dodé et al., 2006). The PCR products were sequenced with the Big Dye terminator kit (Applied Biosystems, Foster City, CA, United States) using one of the PCR primers on an ABI prism 3,130 DNA Genetic Analyzer (Applied Biosystems) in accordance with the recommendations of the manufacturer.

### *In-silico* Analysis

To gain insight into the effect of the newly identified genetic variation p.(Lys205del), we carried out an *in silico* analysis. We first tested the impact of genetic variation on splicing by creating or abolishing a splice site. This analysis was performed using the Human Splicing Finder program (Desmet et al., 2009)^1^. We then carried out an *in silico* 3D structure prediction using Phre2 tool (Kelley et al., 2009)^2^ in order to predict the effect of genetic variation on protein folding.

### Exome Analysis

An in-house pipeline analysis was used to generate VCF files. Then, the annotation and the prioritization of variants were carried out using VarAFT tool, version 2.04^3^. A disease-causing gene approach was used to extract variants located in 46 CHH-related genes extracted from the Online Mendelian Inheritance in Men database and recent literature (Supplementary Table 1). Functionally relevant variants located in exonic genomic regions and splice sites were then selected from the list of variants contained in the VCF file. Variants located in the regulatory regions (UTR, promoters, enhancers, and miRNA binding sites, etc.) flanking the *PROKR2* gene were also screened. Regulatory regions were retrieved using the Genome Browser tool provided by the University of California Santa Cruz database^4^ as well as the GeneCards Human Gene database^5^. The functional effects of the genetic sequence variants were evaluated by *in silico* prediction tools including SIFT^6^, PolyPhen^7^, FATHMM^8^, MutationTaster^9^, MutationAssessor 1.0^10^, PROVEAN v1.1^11^, and Varsome^12^. Furthermore, in order to pinpoint the variants that could potentially be associated with disease expression in the index cases (HH1 and HH12), each selected variant of interest was compared to the exome data of 92 unrelated Tunisian individuals stored in the local database. This approach allowed us to avoid bias especially that public databases, such as ESP6500, GnomAD, or 1000Genomes, do not contain relevant information regarding the genetic background of individuals from the Middle East, North Africa populations.

### Digenic Effect Prediction

The Oligogenic Resource for Variant Analysis (ORVAL) online software^13^ was used to predict the effect of variant combinations in disease-causing genes. This tool is based on variant annotation and effect prediction of two predictive methods named VarCOPP and Digenic Effect predictor. The evaluation scores of digenic combinations include the support score (SS) and the classification score (CS). SS informs about the percentage of algorithms integrated in VarCOPP and Digenic Effect predictor that support the pathogenicity of a given bi-locus combination. Its value ranges from 0 to 100. For candidate disease-causing combinations, SS should be equal or greater than 50%. The CS should correspond to the median probability of a digenic combination to be disease-causing. Its value ranges from 0 to 1. Variant pairs are considered pathogenic when CS is greater than 0.489. To estimate the probability that a predicted disease-causing combination is a true positive, VarCOPP encompasses 95 and 99% confidence intervals which are delimited by minimal CS and SS values. The 95% confidence interval requires CS and SS values greater than or equal to 0.55 and 75, respectively. A digenic combination falls inside the 99% confidence interval when CS is greater than 0.74 and SS is equal to 100.

The variant combinations were categorized into three classes. The first one, termed “true digenic” involves two variants in two different genes to induce disease expression. Otherwise, if an individual carries only one variant, he will be considered unaffected. The second class is referred as “composite class.” In this case, an individual carrying only one variant expresses the disease, whereas the second genetic locus is a genetic modifier that modifies the severity of the clinical presentation or the age at onset. The third class, called “dual molecular diagnosis,” requires the presence of variants in two different genes inducing two independent clinical entities (Papadimitriou et al., 2019). Only the pathogenic bi-locus combinations for each individual were extracted from the list of variant pairs. Considering that variants found in common between the three HH1 family members would not be at the origin of disease expression, the digenic combinations that were specific to the index case were selected. The specificity criteria were related to the presence or absence of a variant or to its zygosity state, i.e., variants that are homozygous in the index case and heterozygous in the two asymptomatic family members.

## Results

### Clinical Investigation

**FIGURE 1:**
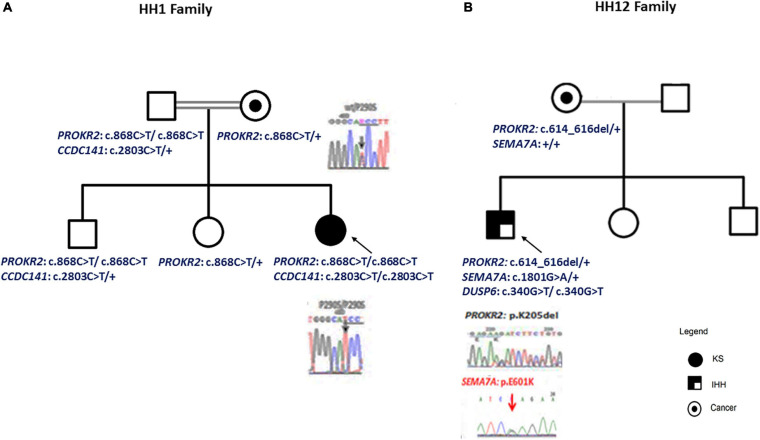
Patient pedigrees and chromatograms of the confirmed mutations. The nuclear family of HH1 **(A)** and HH12 **(B)** patients. Chromatograms of the confirmed mutations are shown.

HH1 is a 21-year-old woman born to first-cousin phenotypically normal parents (Figure 1) and is originating from Central Tunisia. The patient presented congenital anosmia associated with obesity and absence of spontaneous puberty. Her basal and stimulated GnRH–gonadotropin levels were flat, and her MRI of the hypothalamic–pituitary region showed an aplasia of the olfactory bulbs (Table 1). The 17-beta estradiol levels were not measured for this patient. Her family presents a history of lung cancer, congenital deafness, and colon polyps. Except for the cousin of the index case who had delayed puberty, the other family members had normal pubertal development and olfactory tests. HH12 is a 28-year-old man born to unrelated phenotypically normal parents (Figure 1). The patient suffers from nIHH associated with hypertension and obesity. He first consulted for impuberism. A clinical examination showed that his height is 1.78 m and his weight is 113 kg. The genitalia examination showed a micropenis with a gonadal volume of 3 ml. His GnRH response showed a normal response of follicle-stimulating hormone and luteinizing hormone. The testosterone serum level was 1.9 nmol/l (10–41.5 nmol/l). He presents a normal sense of smell and a normal MRI.

**TABLE 1:** Clinical and hormonal features of HH1 and HH12 patients.

						**LH (IU/L)**		**FSH (IU/L)**		
**Case**	**Age (year)**	**Sex**	**Diagnosis**	**Associated clinical features**	**BMI (kg/m^2^**)	**Basal**	**Peak**	**Basal**	**Peak**	**T (nmol/L)**
HH1	21	F	KS	Overweight	25.3	<0.11	0.5	0.11	1.5	
HH12	28	M	nIHH	Hypertension and obesity	35.7	0.7	14.3	0.69	3.4	1.9

*LH, luteinizing hormone; FSH, follicle-stimulating hormone; BMI, body mass index; T, testosterone; IU/L, international unit per liter.*

### Molecular Analysis of the *PROK2* and *PROKR2* Genes

For the HH1 family, after having identified a missense genetic variation, c.868C > T; p.(Pro290Ser), in the *PROKR2* gene at a homozygous state in the proband, we sequenced this gene in the relatives: the mother and the sister carried the p.(Pro290Ser) genetic variation at a heterozygous state, but, intriguingly, the father and the brother were homozygous. To confirm these molecular findings, blood sampling, PCR, and sequencing reactions were performed twice. To exclude contamination and paternity problems, all members of this family were genotyped using the identifier kit (Applied Biosystems). The analysis of allele segregation was in favor of paternity inclusion.

**FIGURE 2:**
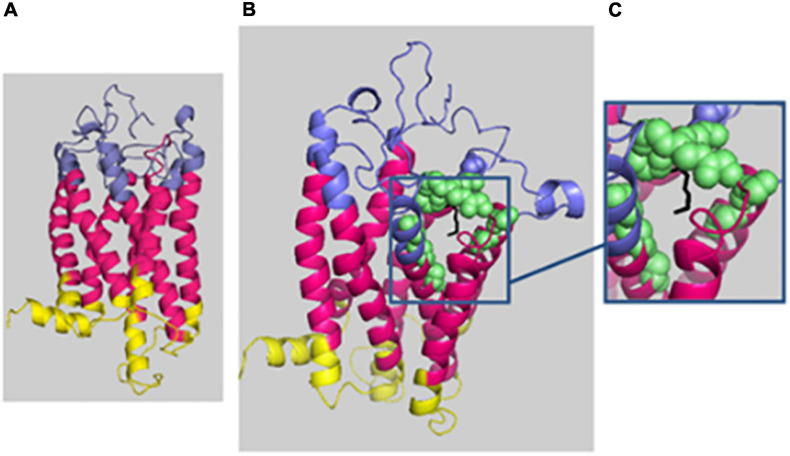
Phyre2 prediction of the deleterious effect of p.(Lys205del) on the 3D protein structure. The protein backbone is shown in cartoon representation, with the extracellular domain (EC) in blue, the transmembrane domain (TM) in hot pink, and the intracellular domain in yellow. **(A)** Mutated protein prediction. **(B)** WT protein prediction. **(C)** Zoomed-in image of residue 205 (localized in EC3) shown as black sticks, and the residue interactions around 4 Å are shown as green spheres: 131N and 136T (EC 2); 193T, 194E, 195T, 204E, 206E, 207F, and 223Y (EC3); and 140Y (TM3) and 227I (TM5). The figure was produced with Pymol.

For HH12, the molecular analysis allowed the identification of a novel variation, c.614_616del; p.(Lys205del), in the *PROKR2* gene at a heterozygous state. Bioinformatics analysis using the Uniprot database showed that the lysine amino acid at position 205 is buried amid the second extracellular loop and that this residue is highly conserved in Prokr2 from mouse, rat, chimp, dog, cow, *Xenopus tropicalis*, and zebrafish and in the human PROKR1 protein. The deleterious effect of this genetic variation could be explained by the creation of a new acceptor splice site as illustrated by the results of the Human Splicing Finder and Genetic Variation Taster tools (donor gained, score = 0.83mut/=0.55 C). The prediction of the p.(Lys205del) variation effect performed with Phyre2 showed a deleterious effect on the 3D protein structure. The comparison between normal and mutant 3D structures was consistent with the deleterious effect. Indeed the lysine amino acid at position 205 interacts with other residues in the EC2, EC3, the third transmembrane domain (TM3), and the TM; thus, its deletion could reduce the PROKR2 protein stability (Figure 2). The genetic investigation of the mother of HH12 showed that she was a carrier of the same likely pathogenic mutation [p.(Lys205del)] at a heterozygous state.

### Exome Analysis and Digenic Effect Prediction for the HH1 Family

**FIGURE 3:**
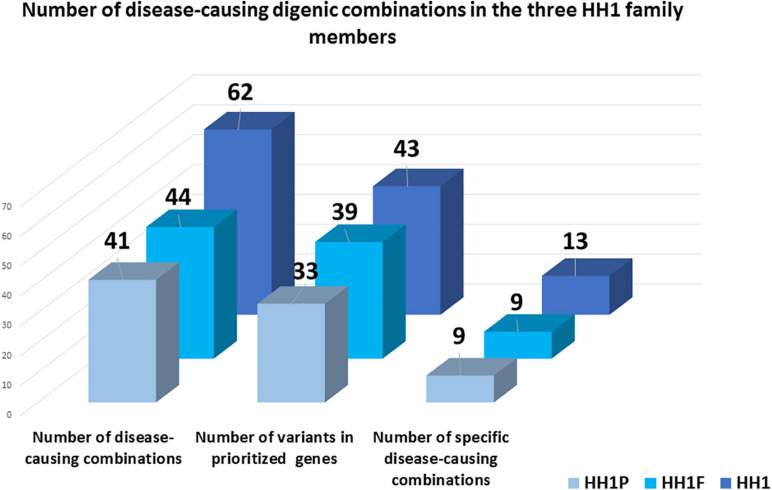
Schematic representation of disease-causing digenic combinations in the three HH1 family members. The histograms correspond to the number of variants in prioritized genes, the total number of pathogenic digenic combinations, and the number of specific pathogenic variant pairs identified in the patient HH1, her asymptomatic brother HH1F, and her father HH1P.

In total, 43, 39, and 33 genetic variants were identified in HH1, her brother, and her father, respectively. For HH1, seven rare variants were identified with a minor allele frequency (MAF) < 0.1 according to the 1000Genomes and GnomAD databases. Four rare variants were harbored by the brother and the father. Besides the p.(Pro290Ser) in the *PROKR2* gene, the remaining rare variants in each case were predicted as benign or as VUS by Varsome search engine as well as 10 *in silico* prediction programs (Supplementary Tables 2–4). For each set of variants identified in the three family members, we proceeded to the digenic effect prediction of variant pairs using the ORVAL software. A total of 940, 828, and 668 digenic combinations were, respectively, obtained in the index case HH1, the asymptomatic brother HH1F, and the father HH1P. In the proband, 62 variant pairs were predicted as pathogenic (Supplementary Table 5), whereas 44 (Supplementary Table 6) and 41 (Supplementary Table 7) disease-causing combinations were found in the brother and father of HH1, respectively (Figure 3). The confidence intervals supporting the pathogenicity of the digenic combinations ranged from 90 to 99%.

**FIGURE 4:**
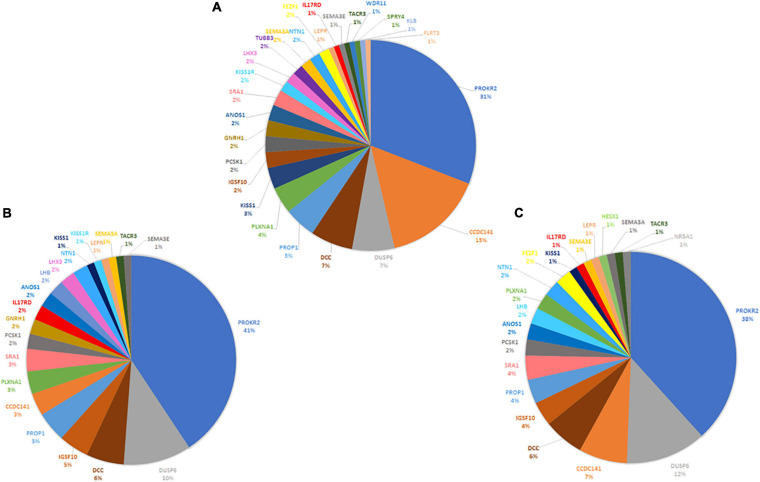
Contribution of CHH-related genes in the digenic inheritance in HH1 family members. Number of disease-causing digenic combinations per gene identified in the index case HH1 **(A)** in the symptomatic brother HH1F **(B)** and in the asymptomatic father HH1P **(C)**.

The three family members carried 19 variants in 10 prioritized genes: *PROKR2*, *DUSP6*, *NTN1*, *ANOS1*, *KISS1R*, *PROP1*, *DCC*, *IGSF10*, *SRA1*, and *PLXNA1*. They shared almost 30 pathogenic digenic combinations involving these variants. In the case of HH1, the *PROKR2* variant [p.(Pro290Ser)] yielded the highest number of bi-locus combinations (31%) predicted as disease-causing, with 25 genes and a median pathogenicity score of 0.62. We also found 13 pathogenic digenic combinations (Figure 3) involving variants that were either only carried by HH1 or those that were homozygous in HH1 and heterozygous in the asymptomatic cases (Table 2). These specific variants included four exonic missense variants in *PCSK1* (NM_000439), *IL17RD* (NM_017563), and *CCDC141* (NM_173648) genes, which were homozygous in the proband and heterozygous in the unaffected family members. Another exonic variant in the *FLRT3* gene (NM_013281), as well as two splice site variants in *WDR11* (NM_018117) and *SMCHD1* (NM_015295) genes, was only present in the index case HH1 (Table 2). All these variants were frequent, with a MAF ranging from 0.2 to 0.6 according to the GnomAD database, except for the missense variant [c.2803C > T; p.(Arg935Trp)] in the *CCDC141* gene (MAF = 0.06) (Supplementary Table 2). This variant was absent in the in-house control database. Despite being predicted as benign by Varsome, this variant [p.(Arg935Trp)] was involved in the second most important pathogenic combinations (15%; 13 genes) in HH1, after the *PROKR2* gene variant [p.(Pro290Ser)]. On the other hand, when the *CCDC141* variant was at a heterozygous state, prediction by ORVAL yielded only two pathogenic digenic combinations with *PROKR2* and *DUSP6* variants in HH1F and HH1P cases (Figure 4). The variant pair *CCDC141* (c.2803C > T)–*PROKR2* (c.868C > T) was classified by ORVAL as true digenic. The contribution of the three missense variants in *IL17RD* and *PCSK1* genes, which were homozygous in the index case and heterozygous in the asymptomatic cases (Table 2), to the total number of pathogenic digenic combinations did not differ among the three family members (Figure 4). This indicates their minor contribution to disease expression in HH1. The splice site variant (c.5476 + 10A > G) in the *SMCDH1* gene (NM_015295), only present in the index case (Table 2), was not involved in any disease-causing digenic combination. The two other genetic variations identified in *FLRT3* (NM_013281) and *WDR11* (NM_018117) genes, present only in HH1 case, represented only 2% of the overall disease-causing variant pairs.

**TABLE 2:** Disease-causing digenic combinations involving specific variants found in the three HH1 family members.

**HH1**								

**Gene_A**	**Gene_B**	**GeneA_Alleles (genomic location)**	**GeneB_Alleles (genomic location)**	**Classification _score**	**Support_score**	**Predicted_class**	**Confidenc _zone**	**Digenic effect class**
*PROKR2*	*PCSK1*	20:5282973:G:A	5:95728974:G:C	0.79	100	Disease-causing	95%-zone	Dual molecular diagnosis
*PROKR2*	*CCDC141*	20:5282973:G:A	2:179721046:G:A	0.77	100	Disease-causing	95%-zone	True digenic
*TUBB3*	*PROKR2*	16:90002290:T:C	20:5282973:G:A	0.73	99.8	Disease-causing	95%-zone	True digenic
*PROKR2*	*KISS1*	20:5282973:G:A	1:204159787:G:C	0.71	98.8	Disease-causing	95%-zone	True digenic
*PROKR2*	*KISS1*	20:5282973:G:A	1:204159922:T:C	0.7	98.8	Disease-causing	95%-zone	True digenic
*TACR3*	*PROKR2*	4:104510766:G:A	20:5282973:G:A	0.62	87.2	Disease-causing	90%-zone	True digenic
*CCDC141*	*KISS1*	2:179721046:G:A	1:204159787:G:C	0.6	76.4	Disease-causing	90%-zone	True digenic
*CCDC141*	*KISS1*	2:179721046:G:A	1:204159922:T:C	0.59	75	Disease-causing	90%-zone	True digenic
*TUBB3*	*CCDC141*	16:90002290:T:C	2:179721046:G:A	0.57	69.39	Disease-causing	90%-zone	True digenic
*PROKR2*	*IL17RD*	20:5282973:G:A	3:57138419:G:A	0.56	65.6	Disease-causing	90%-zone	True digenic
*WDR11*	*PROKR2*	10:122618148:G:C	20:5282973:G:A	0.56	64.4	Disease-causing	90%-zone	True digenic
*SPRY4*	*PROKR2*	5:141693593:C:T	20:5282973:G:A	0.56	64.4	Disease-causing	90%-zone	True digenic
*PROKR2*	*FLRT3*	20:5282973:G:A	20:14306953:G:T	0.54	51.8	Disease-causing	90%-zone	Dual molecular diagnosis

**HH1F**								

*PROKR2*	*PCSK1*	20:5282973:G:A	5:95728974:G:C	0.79	100	Disease-causing	95%-zone	Dual molecular diagnosis
*PROKR2*	*CCDC141*	20:5282973:G:A	2:179721046:G:A	0.74	99.6	Disease-causing	95%-zone	True digenic
*SEMA3A*	*PROKR2*	7:83606518:G:A	20:5282973:G:A	0.7	97.39	Disease-causing	95%-zone	True digenic
*TACR3*	*PROKR2*	4:104510766:G:A	20:5282973:G:A	0.62	87	Disease-causing	90%-zone	True digenic
*PROKR2*	*IGSF10*	20:5282973:G:A	3:151163138:G:A	0.57	68	Disease-causing	90%-zone	True digenic
*PROKR2*	*IL17RD*	20:5282973:G:A	3:57138419:G:A	0.56	64.8	Disease-causing	90%-zone	True digenic
*PROKR2*	*IL17RD*	20:5282973:G:A	3:57136585:C:T	0.55	60.19	Disease-causing	90%-zone	True digenic
*PROKR2*	*SEMA3E*	20:5282973:G:A	7:82996831:A:T	0.55	58.4	Disease-causing	90%-zone	True digenic
*PROKR2*	*CCDC141*	20:5282973:G:A	2:179753245:C:T	0.53	50.4	Disease-causing	90%-zone	True digenic

**HH1P**								

*PROKR2*	*PCSK1*	20:5282973:G:A	5:95728974:G:C	0.79	100	Disease-causing	95%-zone	Dual molecular diagnosis
*PROKR2*	*HESX1*	20:5282973:G:A	3:57232504:T:C	0.76	100	Disease-causing	95%-zone	True digenic
*PROKR2*	*CCDC141*	20:5282973:G:A	2:179721046:G:A	0.74	99.6	Disease-causing	95%-zone	True digenic
*NTN1*	*PROKR2*	17:9086293:G:C	20:5282973:G:A	0.65	91	Disease-causing	95%-zone	True digenic
*TACR3*	*PROKR2*	4:104510766:G:A	20:5282973:G:A	0.62	87	Disease-causing	90%-zone	True digenic
*NR5A1*	*PROKR2*	9:127262802:C:G	20:5282973:G:A	0.59	74.8	Disease-causing	90%-zone	True digenic
*PROKR2*	*IL17RD*	20:5282973:G:A	3:57138419:G:A	0.56	64.8	Disease-causing	90%-zone	True digenic
*FEZF1*	*PROKR2*	7:121942847:G:C	20:5282973:G:A	0.56	60.8	Disease-causing	90%-zone	True digenic
*PROKR2*	*CCDC141*	20:5282973:G:A	2:179753245:C:T	0.53	50.4	Disease-causing	90%-zone	True digenic

We also evaluated the involvement of other variants located in the flanking regulatory regions of the *PROKR2* gene, which could be responsible for the incomplete penetrance in the family members of HH1. On the basis of predictions provided by the Encyclopedia of DNA Elements and the GeneHancer database, we focused on regulatory regions adjacent to the *PROKR2* gene, likely covered by whole-exome sequencing (WES). The closest promoter and enhancer regions are located in the exon 1–intron 1 junction of *PROKR2*. This region is rich with CpG islands and overlaps with the *AX746654* gene. Two transcription factors, namely, HDAC2 and CTCF, are known to bind to this region. The WES analysis did not reveal any variants in the regulatory region of *PROKR2* nor in the genes encoding its transcription factors.

### Exome Analysis and Digenic Effect Prediction for the HH12 Family

For HH12, 35 variants were found in 25 prioritized genes. Three variants in three genes were rare, including the *PROKR2* gene mutation [p.(Lys205del)], a novel heterozygous missense variant [c.1801G > A; p.(Glu436Lys)] in the *SEMA7A* gene (NM_001146029), as well as a splice site variation in the *PLXNA1* gene (NM_032242; MAF = 0.03 in GnomAD). The *SEMA7A* gene variant was predicted as a VUS according to Varsome, whereas the *PLXNA1* gene variation was classified as benign. The *SEMA7A* variant [p.(Glu436Lys)] was absent in the 92 exomes of our local database (Supplementary Table 8).

The ORVAL prediction revealed five pathogenic variant pairs (confidence interval = 90–95%) involving *DUSP6*, *ANOS1*, *DCC*, *PROP1*, *PLXNA1*, and *SEMA7A* genes (Table 3 and Supplementary Table 9). On the other hand, no disease-causing digenic combinations included the *PROKR2* gene variant p.(Lys205del). The *DUSP6* gene [c.340G > T; p.(Val114Leu)] was involved in all five disease-causing digenic combinations. Sanger sequencing showed that the *SEMA7A* variant [c.1759G > A; p.(Glu587Lys)] was only present in HH12 and absent in his asymptomatic mother (Figure 1). The variants located in the promoter region of *PROKR2* were extracted, which revealed one common variant (c.−9 + 342A > G) in intron 1 with a MAF of 0.3 according to GnomAD. Varsome is the only prediction tool that annotated this variant as benign. We have also identified in the *CTCF* gene, predicted as a transcription factor of the *PROKR2* gene, one variant (c.^∗^29T > G) which is also frequent (MAF = 0.1) and predicted as benign by Varsome. Furthermore, the ORVAL prediction tool was employed to evaluate the digenic effect of both variants in combination with the p.(Lys205del) identified in *PROKR2* gene. However, no digenic combinations were identified.

**TABLE 3:** Disease-causing digenic combinations identified in HH12.

**Gene_A**	**Gene_B**	**GeneA_Alleles (genomic location)**	**GeneB_Alleles (genomic location)**	**Classification _score**	**Support _score**	**Predicted_class**	**Confidence_zone**	**Digenic_effect_class**
*DUSP6*	*ANOS1*	12:89745477:C:A	X:8504833:C:T	0.68	96.8	Disease-causing	95% zone	True digenic
*PLXNA1*	*DUSP6*	3:126751689:G:A	12:89745477:C:A	0.54	52.8	Disease-causing	90% zone	Monogenic + modifier
*DCC*	*DUSP6*	18:49867224:T:C	12:89745477:C:A	0.66	94.8	Disease-causing	95% zone	Dual molecular diagnosis
*SEMA7A*	*DUSP6*	15:74703165:C:T	12:89745477:C:A	0.59	74	Disease-causing	90% zone	Dual molecular diagnosis
*PROP1*	*DUSP6*	5:177422823:C:T	12:89745477:C:A	0.58	70.8	Disease-causing	90% zone	?

**TABLE 4:** Clinical characteristics of patients having a monoallelic or a biallelic p.(Pro290Ser) mutation.

**References**	**Gender cases**	**Genotype**	**Familial**	**BMI**	**Sense of**	**Olfactory**	**Cryptorchidism/**	**Spontaneous**	**LH (IU/L)**	**FSH (IU/L)**
			**cases**		**smell**	**bulb MRI**	**microphallus**	**puberty**	**Basal-peak**	**Basal-peak**
Sarfati et al., 2010a,b	M	p.(Pro290Ser)/ +	No	22.5	Anosmia	NA	No/no	No	0.1–0.1	0.2–1.2
	M	p.(Pro290Ser)/ +	No	28	Hyposmia	NA	No/no	No	0.7–10.5	0.7–2.4
	F	p.(Pro290Ser)/ +	Yes	NA	Hyposmia	NA		Partial breast development	4–NA	4–NA
	F	p.(Pro290Ser)/p.(Pro290Ser)	No	24.3	Anosmia	Hypoplasia		No	0.1–0.5	0.2–1.5
	F	p.(Pro290Ser)/p.(Pro290Ser)	No	21.2	Anosmia	Aplasia		No	0.1–2.2	0.6–4.0
Dodé et al., 2006	M	p.(Pro290Ser)/ +	No	28.3	Anosmia	Aplasia	Yes/no	No	0.2–0.5	0.5–2
	M	p.(Pro290Ser)/ +	No	20.3	Hyposmia	_	Yes/yes	No	0.3–1.3	0.2–3.4
Present study	F	p.(Pro290Ser)/p.(Pro290Ser)	Yes	25.3	Anosmia	Hypoplasia		No	>0.11–0.5	0.11–1.5

*LH, luteinizing hormone; FSH, follicle-stimulating hormone; IU/L, international unit per liter; NA, not applicable.*

## Discussion

**FIGURE 5:**
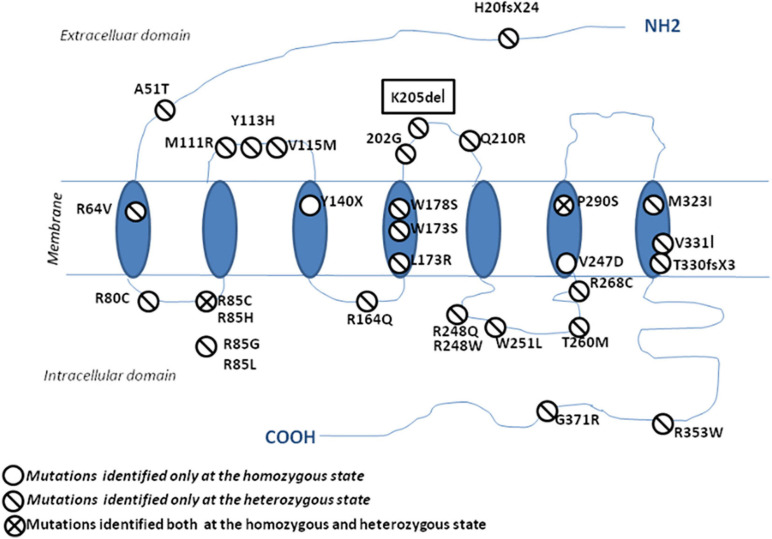
Schematic representation of the protein localization of *PROKR2* genetic variations. The genetic variations identified at a heterozygous state are shown with a one-bar circle, the genetic variations identified at both homozygous and heterozygous states are shown with a two-bar circle, and the genetic variations identified only at a homozygous state are shown with an empty circle.

Since 2006, many studies have reported *PROK2/PROKR2* genetic variations mainly missense, nonsense, and frameshift genetic alterations (Figure 5; Sarfati et al., 2010b; Sbai et al., 2014). Most patients carry monoallelic genetic variations, but some patients may carry bi-allelic variants either in the same or in different genes, indicating a dominant, recessive, or oligogenic inheritance, respectively (Dodé et al., 2006; Pitteloud et al., 2007; Leroy et al., 2008; Sarfati et al., 2010b). The p.(Pro290Ser) variant in *PROKR2* gene has been described in CHH patients at heterozygous (Dodé et al., 2006) and homozygous (Sarfati et al., 2010a) states and is always associated with clinical presentations similar to those seen in HH1 (Table 4). The p.(Pro290Ser) mutation is located in the sixth transmembrane (TM) domain. TM domains are important for ligand binding and receptor trafficking (Tan et al., 2004). Functional assays predicted that this genetic variation impairs not only cell surface-targeting of the receptor but also its ability to activate the Gq Protein (Monnier et al., 2009). In the present study, p.(Pro290Ser) in the *PROKR2* gene was identified both in the proband and her healthy father and brother, all at homozygous states. The identification of homozygous deleterious genetic variations among healthy relatives has been previously described for diverse genetic diseases (Bonyadi et al., 2009; Biegstraaten et al., 2011; Suk et al., 2011). The question that arises is how to explain that such a deleterious genetic variation could be seen in unaffected relatives. The most plausible explanations are (i) possible digenic or oligogenic interactions due to the co-occurrence of additional genetic variants (Sykiotis et al., 2010) and (ii) the involvement of epigenetic factors (Abreu et al., 2010; Dode and Hardelin, 2010; Noel and Kaiser, 2011; McCabe et al., 2013).

Several studies employing next-generation sequencing have revealed that more and more variations known to be causative of genetic diseases can also be found among controls, thus further demonstrating the complexity of heredity (Olson, 2008; Klassen et al., 2011; Nishiguchi and Rivolta, 2012). Hence, the correlation between oligogenicity and the presence of a severe phenotype is often unclear. Sykiotis et al. (2010) reported that the same CHH phenotype seen in a propositus, carrying digenic genetic variations in *FGFR1* and *PROKR2* genes, was also observed in another family member who harbored only the genetic variation in the *FGFR1* gene. This finding proves that carrying more or fewer genetic variations may not always correlate with the severity of disease expression, which further demonstrates the incomplete penetrance of many suspected variants.

Following exome sequencing in the family of HH1, we identified 42 additional variants in 25 candidate genes in the index case as well as 39 and 33 variants in her asymptomatic brother and father, respectively. The disease-causing digenic combination profiles between the HH1 family members were compared (Figure 4). The results showed that, in addition to the *PROKR2* gene variant [p.(Pro290Ser)], a second variant in c.2803C > T in the *CCDC141* gene was involved in the second highest number of pathogenic digenic combinations (15%), with 18 other variants in 13 genes. The *CCDC141* variant was found at a homozygous state in the patient HH1 and at a heterozygous state in the asymptomatic cases. Our analysis indicated that the zygosity state of the c.2803C > T variant in the *CCDC141* gene considerably influenced the rate of pathogenic combinations. The same variant, when heterozygous in the asymptomatic cases, contributed to only 3% of the total number of pathogenic digenic combinations (Figure 4). In the same context, the implication of the *IL17RD* and *PCSK1* variants in inducing the expression of CHH was ruled out in our propositus as they took part in the same number of digenic combinations with similar classification scores in all three family cases (Figure 4). The *CCDC141* gene encodes for a coiled-coil domain-containing protein whose function is not clearly established. In mouse models, it has been shown that ccdc141 is expressed in GnRH neurons and olfactory fibers. The role of *CCDC141* in the embryonic migration of GnRH neurons has been highlighted in human patients and mouse models (Hutchins et al., 2016). The prevalence of *CCDC141* variants in HH patients was estimated to be 3.3% in the cohort studied by Turan et al. (2017). A nonsense variant p.(Arg724^∗^) was identified in 20 probands with KS along with a loss-of-function variant in the *FEZF1* gene (Hutchins et al., 2016). In 75% of the reported families, at least one additional likely pathogenic mutation in another causative gene was identified, hence underscoring the oligogenic involvement of *CCDC141* gene variants in CHH (Turan et al., 2017). The c.2803C > T variant identified in the HH1 family has not been previously associated with CHH. However, it has previously been annotated as damaging in genome-wide association studies (Marsman et al., 2014; van den Berg et al., 2017; Lin et al., 2018). In light of the *in silico* digenic effect prediction, we hypothesize that its implication in 18 disease-causing combinations, compared to only two pathogenic variant pairs in the healthy family members, may be at the origin of disease expression in the index case (HH1), while suggesting that the full penetrance of the *CCDC141* gene variant is associated to an autosomal recessive inheritance mode. It is also worth noting that the brain structure and function present differences between males and females. More than 2,000 genes show expression divergence between the two sexes at all developmental stages, especially during puberty (Shi et al., 2016). This may suggest that the variable expressivity in the HH1 family might be, in part, related to the different sex-specific gene expression profiles between our female patient and the asymptomatic male family members.

For the HH12 family, the healthy mother and her affected son were both heterozygous for the p.(Lys205del) variation in the *PROKR2* gene. The same situation was previously described among several patients (Dodé et al., 2006; Abreu et al., 2008; Cole et al., 2008; Sykiotis et al., 2010). The WES analysis revealed a total of 33 variants [33 variants in coding regions/splice sites (three rare); five in UTR] in 28 genes. The ORVAL prediction revealed five disease-causing digenic combinations involving *DUSP6*, *ANOS1*, *DCC*, *PLXNA1*, *PROP1*, and *SEMA7A* genes (Table 3). The absence of variant combinations involving the *PROKR2* gene variant p.(Lys205del) excludes its implication in digenic inheritance in the index case (HH12). Furthermore, since the variant is novel and has no functional evidence of pathogenicity, it is likely to be benign. Further molecular studies are needed to prove the deleterious character of the *PROKR2* Lys205del variant.

Except for the *SEMA7A* gene variant [p.(Glu436Lys)], mutations identified in *DUSP6*, *ANOS1*, *DCC*, *PLXNA1*, and *PROP1* genes were carried by HH1 family cases (HH1, HH1F, and HH1P) and involved in pathogenic digenic combinations with the *DUSP6* gene variant [p.(Val114Leu)]. Such findings bring into question their involvement in disease expression in HH12. The *SEMA7A* variant [p.(Glu436Lys)] was predicted as VUS by Varsome. Sanger validation revealed the absence of this mutation in the healthy mother. The *SEMA7A* and *DUSP6* genes were implicated in a digenic combination classified as “dual molecular diagnosis” by ORVAL. We hypothesize that the disease expression in HH12 may be explained by the digenic transmission of the *SEMA7A* and *DUSP6* variants or a monogenic inheritance involving only the *SEMA7A* VUS if further functional assays would allow its reclassification into pathogenic.

## Conclusion

The presence of homozygous *PROKR2* deleterious genetic variations in asymptomatic first-degree relatives and siblings is apparently insufficient to cause KS/nIHH. This strongly suggests the incomplete penetrance trait of the disease. Further investigations are required to clarify the involvement of additional genetic and environmental factors in GnRH deficiency. Exome data analysis allowed us to explore the implication of oligogenism as a possible mechanism involved in the incomplete penetrance witnessed in the investigated families. Nonetheless, the molecular mechanisms that modulate the oligogenic interactions are far from being elucidated since the exact roles of some susceptibility genes in the physiological regulation of the GnRH nervous system are yet to be discovered.

## Data Availability Statement

Processed data related to Sanger sequencing and digenic combinations are available in the article. Raw data related to WES are available from the corresponding author upon reasonable request. Note that in Tunisia, genetic data are considered as personal private data, therefore the minimal dataset was submitted as Supplementary Material. The complete raw data may be made available upon request after obtaining IRB approval.

## Ethics Statement

Patients were interviewed by both a clinical investigator and a geneticist using a structured hypogonadotropic hypogonadism questionnaire. Written informed consent was obtained from both patients and controls in accordance with the Declaration of Helsinki and after being approved by the Institutional Review Board (Registration numbers IRB00005445 and FWA00010074).

## Author Contributions

RM contributed to exome analysis, manuscript drafting, molecular investigation, quality control, and digenic score calculation. LCBA contributed to data analysis, manuscript drafting, recruitment of patients, molecular diagnosis, technical supervision, and quality control. SL contributed to functional *in silico* annotation and exome result interpretation. ZT and MS contributed to clinical diagnosis and characterization, and result evaluation. SE and CN contributed to whole exome data analysis and quality control of bioinformatic pipeline. YB contributed to molecular study, technical assistance, and 3D protein structure determination and interpretation. KM and AB contributed to data evaluation and quality control. SA contributed to study design and supervision, quality control, and manuscript revision. OM contributed to study design and supervision, exome data interpretation, quality control, and manuscript drafting and revision. All authors contributed to the article and approved the submitted version.

## Conflict of Interest

The authors declare that the research was conducted in the absence of any commercial or financial relationships that could be construed as a potential conflict of interest.

## Publisher’s Note

All claims expressed in this article are solely those of the authors and do not necessarily represent those of their affiliated organizations, or those of the publisher, the editors and the reviewers. Any product that may be evaluated in this article, or claim that may be made by its manufacturer, is not guaranteed or endorsed by the publisher.

## Footnotes

1
http://www.umd.be/HSF/

2
http://www.sbg.bio.ic.ac.uk/phyre2/html/page.cgi?id=index

3
http://varaft.eu/

4
https://genome.ucsc.edu/

5
https://www.genecards.org/

6
https://sift.bii.a-star.edu.sg/

7
genetics.bwh.harvard

8
http://fathmm.biocompute.org.uk

9
http://mutationtaster.org/

10
http://mutationassessor.org/r3/

11
provean.jcvi.org/

12
https://varsome.com

13
https://orval.ibsquare.be/

## Supplementary Material

Supplementary Table 1 List of CHH-related genes prioritized in the exome analysis for HH1 and HH12 families.

Supplementary Table 2 List of variants identified in prioritized genes in the index case HH1.

Supplementary Table 3 List of variants identified in prioritized genes in the asymptomatic brother HH1F.

Supplementary Table 4 List of variants identified in prioritized genes in the asymptomatic father HH1P.

Supplementary Table 5 List of digenic combinations identified in the index case HH1.

Supplementary Table 6 List of digenic combinations identified in the asymptomatic brother HH1F.

Supplementary Table 7 List of digenic combinations identified in the asymptomatic father HH1P.

Supplementary Table 8 List of variants identified in prioritized genes in the index case HH12.

Supplementary Table 9 List of digenic combinations identified in the index case HH12.
